# The Council of the Royal College of Surgeons

**Published:** 1897-06-19

**Authors:** 


### THE COUNCIL OF THE ROYAL COLLEGE OF SURGEONS.

At an ordinary meeting of the Council of the Royal College of Surgeons of England, Sir William MacCormac, F.R.C.S.Eng., president of the college, in the chair, Sir James Paget, F.R.S., F.R.C.S.Eng., Serjeant-Surgeon to the Queen, and Lord Lister, P.R.S., F.R.C.S. Eng., Surgeon-Extraordinary to the Queen, having been introduced, the President presented to each the honorary medal of the college, together with a document declaratory of the award thereof. Mr. Charles Sissmore Tomes, F.R.S., M.R.C.S.Eng., was introduced and received the John Tomes prize, together with a document declaratory of the award.

After this the council proceeded to the further consideration of a memorial, signed by 41 members of the medical profession resident in Norwich, giving particulars respecting the Norwich Medical Institute, and asking the advice and guidance of the council on the question of meeting in consultation the medical officers of the institute. It was resolved that the following reply be sent: "That the council, having considered the memorial of members of the medical profession in Norwich, and the additional statements made by the deputation from Norwich, are of opinion that the disciplinary powers provided by Section XVI. of the bye-laws do not entitle the council to call in question the legality of 'fellows and members' of the college holding the appointment of medical officer to the Norwich Medical Institute or to similar institutions, and therefore they cannot endorse the refusal of any of its fellows or members to meet in consultation (particularly in urgent cases) registered practitioners when acting in conformity with their legal rights." The council further considered a letter from the committee of the Portsmouth Medical Union, asking whether the college would be prepared (provided suitable evidence was forthcoming) to take action against its members relative to the practice of "touting" and canvassing from door to door carried on by some members who run private medical clubs and who employ a paid collector for the purpose, and also against those members who belong to and hold office in the so-called medical aid societies where canvassers are employed and who "tout" for the benefit of particular individual practitioners. In the case of the private club it was stated the employment of a canvasser is direct; in that of the medical aid society exactly the same evils exist, but the employment of canvassers, so far as the doctor is concerned, is indirect. In both cases the collector or canvasser is paid on commission, and in the case of the medical aid society it is known that some medical men actually bribe the canvassers to "tout" especially for them. It was resolved that the following reply be sent to the letter: "The council, whilst not prepared to express an abstract opinion on the question raised by the Portsmouth Medical Union, are willing to consider any evidence which may be submitted to them respecting canvassing for patients either directly or indirectly by any individual fellow or member of the college, with a view to determining whether or not such fellow or member has been guilty of a breach of the bye-laws."

